# Oocyte-derived E-cadherin acts as a multiple functional factor maintaining the primordial follicle pool in mice

**DOI:** 10.1038/s41419-018-1208-3

**Published:** 2019-02-15

**Authors:** Hao Yan, Jia Wen, Tuo Zhang, Wenying Zheng, Meina He, Kun Huang, Qirui Guo, Qian Chen, Yi Yang, Guangcun Deng, Jinrui Xu, Zhiqing Wei, Hua Zhang, Guoliang Xia, Chao Wang

**Affiliations:** 1 Key Laboratory of Ministry of Education for Conservation and Utilization of Special Biological Resources in the Western China, College of Life Science, NingXia University, Yinchuan, Ningxia 750021 China; 2 State Key Laboratory of Agrobiotechnology, College of Biological Science, China Agricultural University, Beijing, 100193 China

## Abstract

In mammals, female fecundity is determined by the size of the primordial follicle (PF) pool, which is established during the perinatal period. As a non-renewable resource, the preservation of dormant PFs is crucial for sustaining female reproduction throughout life. Although studies have revealed that several oocyte-derived functional genes and pathways, such as newborn ovary homeobox (NOBOX) and 3-phosphoinositide-dependent protein kinase-1, participate in maintaining the PF pool, our understanding of the underlying molecular mechanisms is still incomplete. Here, we demonstrate that E-cadherin (E-cad) plays a crucial role in the maintenance of PFs in mice. E-cad is specifically localized to the cytomembrane of oocytes in PFs. Knockdown of *E-cad* in neonatal ovaries resulted in significant PF loss owing to oocyte apoptosis. In addition, the expression pattern of NOBOX is similar to that of E-cad. Knockdown of *E-cad* resulted in a decreased NOBOX level, whereas overexpression of *Nobox* partially rescued the follicle loss induced by silencing *E-cad*. Furthermore, E-cad governed NOBOX expression by regulating the shuttle protein, β-catenin, which acts as a transcriptional co-activator. Notably, E-cad, which is a transmembrane protein expressed in the oocytes, was also responsible for maintaining the PF structure by facilitating cell–cell adhesive contacts with surrounding pregranulosa cells. In conclusion, E-cad in oocytes of PFs plays an indispensable role in the maintenance of the PF pool by facilitating follicular structural stability and regulating NOBOX expression. These findings shed light on the physiology of sustaining female reproduction.

## Introduction

Ovarian follicles serve as the basic and non-renewable reproductive unit of female mammals^1^. The primordial follicle (PF), which consists of an oocyte and its surrounding flattened somatic cells, is not only the initial stage of follicle development, but is also the dominating resource of ovarian follicles^2,3^. The majority of PFs remain quiescent in the ovary for a long time before they are recruited into the growing pool^4^. In humans, this resting state could last for decades^5^. During this period, the progressive loss of PFs, which is initiated by the oocyte, occurs in the ovary^6^. Therefore, the preservation of dormant PFs is highly important for female reproductive potential and duration. Any aberration probably results in infertility or early exhaustion of follicles^7^.

Oocyte-derived molecules are pivotal for the survival of PFs after birth. It has been reported that 3-phosphoinositide-dependent protein kinase-1, the upstream molecule of protein kinase B (AKT), preserves the reproductive lifespan by maintaining the survival of ovarian PFs^8^. We have demonstrated that transforming growth factor-β signaling participates in the maintenance of the PF pool in mice through regulating tuberous sclerosis complex/mammalian target of rapamycin complex 1 (mTORC1) signaling in oocytes^9^. The phosphatidylinositol 3-kinase (PI3K) and mTORC1 pathways control PF activation and their activators have been applied in the in vitro activation of PFs in the clinic to treat premature ovarian insufficiency (POI), which is generally characterized by amenorrhea before the age of 40 years^2,4,10–12^. In addition, newborn ovary homeobox (NOBOX), which is a transcription factor preferentially expressed in oocytes, is critical for oocyte-specific gene expression and early folliculogenesis^13,14^. Ablation of *Nobox* in mouse ovaries has a limited effect on PF formation. However, the oocytes rarely grow beyond 20 μm and are rapidly lost after birth. Coincidentally, dysregulation of the human homolog of *Nobox* is related to POI^5^. Although the role of above molecules and pathways in controlling the preservation of PFs has been revealed, the detailed mechanism needs further study to better understand the etiology of POI.

Generally, cell adhesion is essential for tissue structure and function^15^. As the indispensable compartments of cell–cell contacts, the cadherin family members play a key role in cell–cell recognition and adhesion and interact with intracytoplasmic proteins through adaptor proteins such as catenins^16–18^. E-cadherin (E-cad), also called cadherin1 (CDH1), is a calcium-dependent cell adhesion molecule that is involved in the establishment and maintenance of epithelial cell morphology during embryogenesis and adulthood^19^. E-cad is defined as a single-pass transmembrane protein that interacts with β-catenin by its cytodomain and attaches to the actin cytoskeleton^20^. Dysregulation of E-cad expression or function disrupts embryonic morphogenesis and alters the characteristics of differentiated cells^19–21^. In addition, E-cad plays roles in signal transduction from the cytomembrane to nucleus via β-catenin, which is not only a transcriptional co-activator but also a well-accepted binding protein of E-cad in cell adhesion^22^. E-cad is involved in multiple ovarian developmental events in mice, such as primordial germ cell migration and germline cyst breakdown before PF formation^23,24^. In addition, E-cad regulates granulosa cell differentiation in preantral follicles^25^. However, the potential role of E-cad in sustaining dormant PFs has not yet been revealed.

The current study shows that oocyte-expressing E-cad perform versatile functions in maintaining PFs in mouse ovaries. Membrane-localized E-cad regulates NOBOX expression by interacting with β-catenin. E-cad also facilitates the establishment of the PF structure by promoting cell–cell contacts between the oocytes and surrounding pregranulosa cells.

## Results

### Oocyte-expressing E-cad is indispensable for maintaining the PF pool

**Fig. 1:**
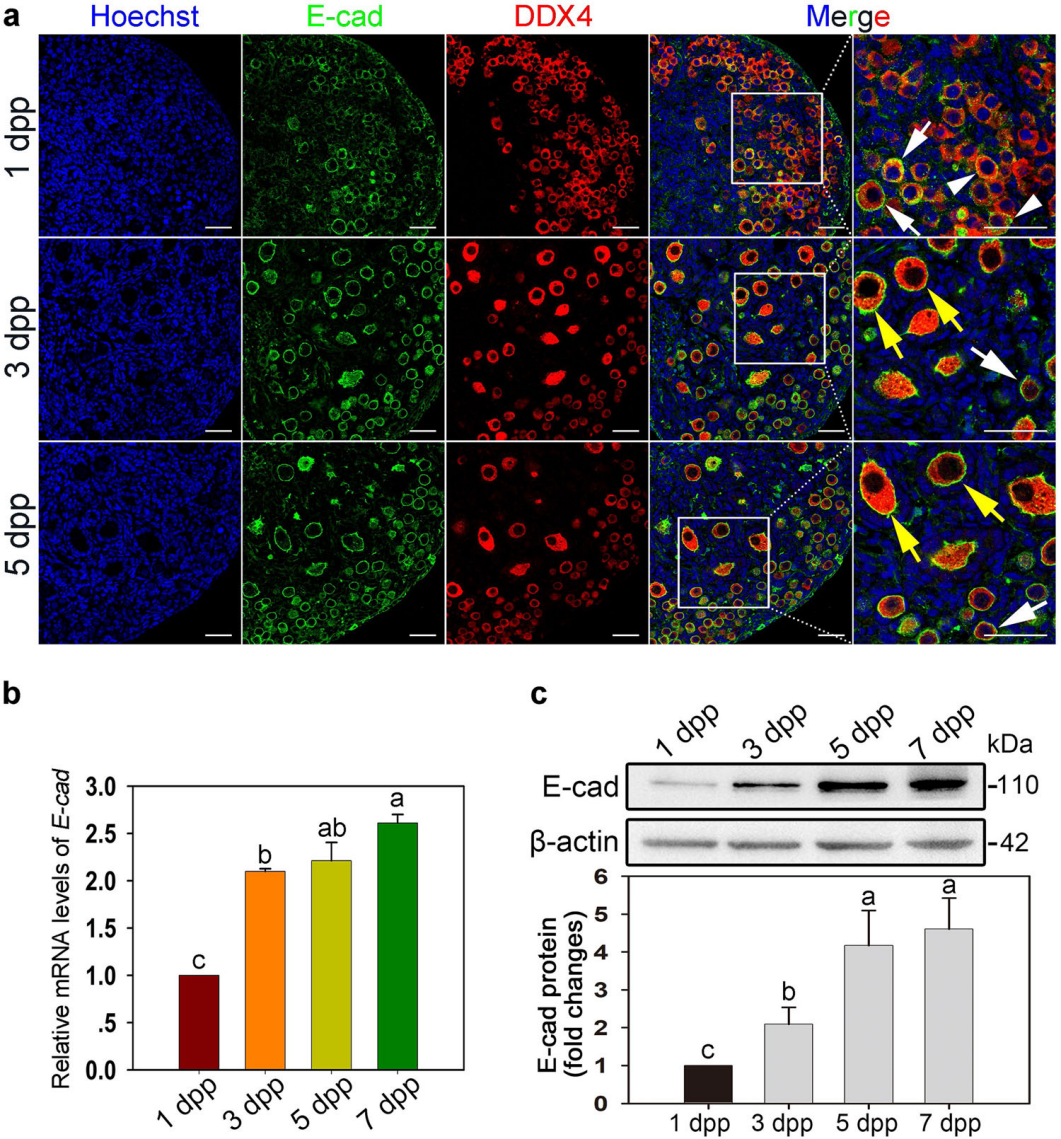
E-cad expression pattern in the neonatal mouse ovaries. **a** Cellular localization of E-cad in ovaries. Ovaries were stained for E-cad (green) and the oocyte marker DDX4 (red) at the indicated time points. The nuclei were counter-stained by Hoechst (blue). E-cad was mainly localized to the cytomembrane of oocytes in both primordial follicles (white arrows) and growing follicles (yellow arrows). **b** qRT-PCR assay showed that *E-cad* mRNA increased at 3 dpp. **c** Western blot assay showed that E-cad protein expression was increasing from 1 dpp to 7 dpp. The experiments were repeated at least three times, and representative images are shown. The data are presented as the means ± S.D. and considered statistically significant at *P* < 0.05. Scale bars: 50 μm

To investigate the potential role of E-cad in early follicular development, immunofluorescence staining was employed to detect the cellular localization and expression dynamics of E-cad in neonatal mouse ovaries. E-cad was localized to the cytomembrane of some germ cells in cysts from 1 day post-partum (dpp) ovaries (Fig. 1a, arrowheads). Along with PF formation, the expression of E-cad was observed in all of the PFs (Fig. 1a, white arrows). From 3 dpp to 7 dpp, the expression of E-cad was increasing in both PFs and growing follicles compared with germ cell cysts (Fig. 1a, yellow arrows). The qRT-PCR and western blot analyses revealed that both the mRNA and protein expression of *E-cad* significantly increased with the establishment of the PF pool at 3 dpp and was retained at a higher level from 5 dpp to 7 dpp, during which PF activation was generally initiated (Fig 1b, c). These results indicate that E-cad plays a potential role in the maintenance and activation of PFs in the mouse ovary.

**Fig. 2:**
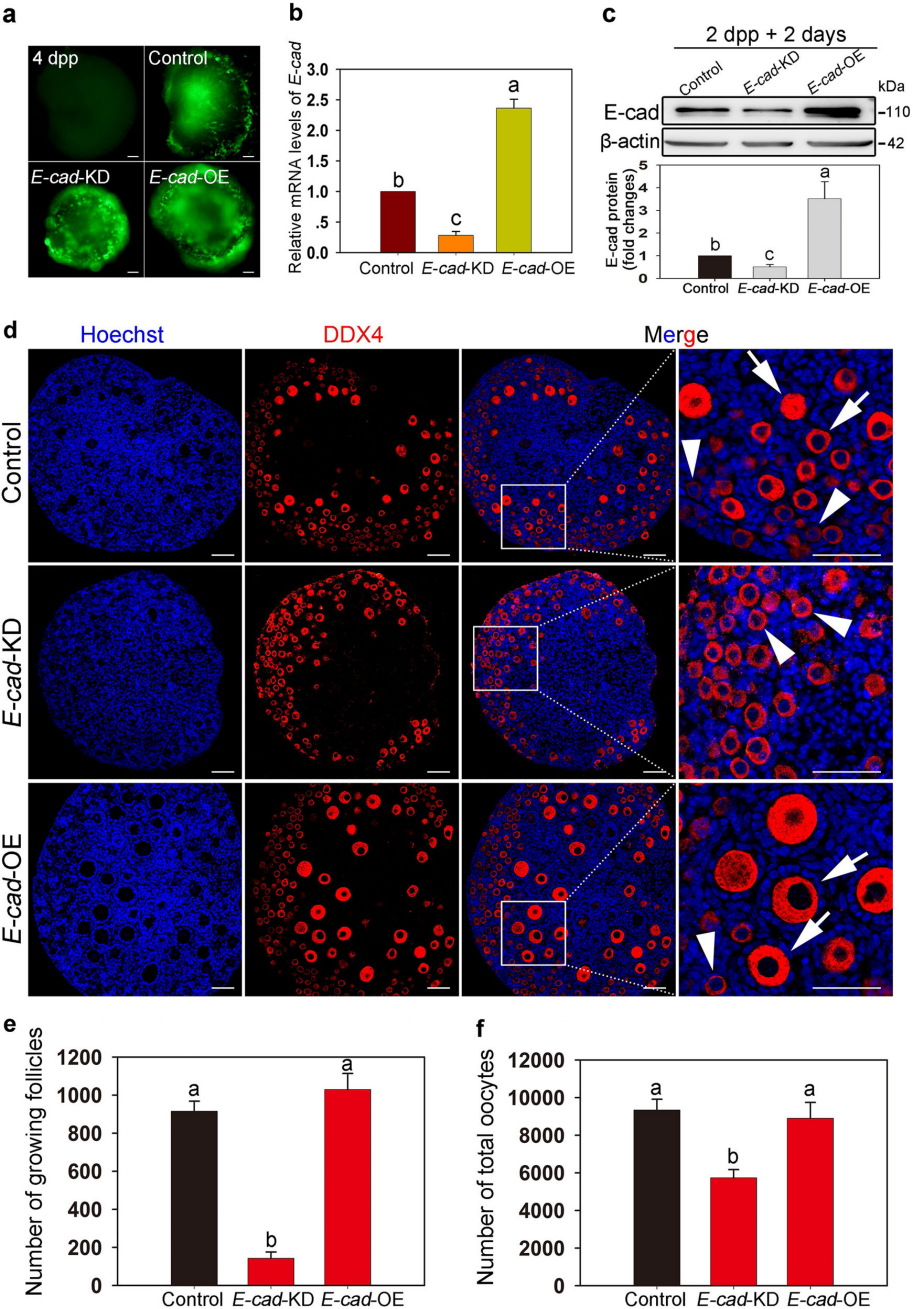
E-cad is involved in maintaining the primordial follicles pool and supports follicle growth in mice. **a** Lentiviral transfection efficiency analysis. The ovaries at 2 dpp were transfected with an empty lentivirus or lentiviral construct expressing *E-cad*-shRNA (*E-cad-*KD) or *E-cad* (*E-cad-*OE) and cultured for 2 days in vitro. Green fluorescence from the GFP reporter was observed in the ovaries, demonstrating the satisfactory efficiency of *E-cad*-KD and *E-cad*-OE transfection. **b**, **c** qRT-PCR and western blot analyses confirmed the efficiency of *E-cad* knockdown and overexpression. Both the mRNA and protein levels of *E-cad* were significantly decreased in *E-cad*-KD group and increased in *E-cad*-OE group. **d** The ovaries at 2 dpp were transfected with indicated lentiviruses and cultured for 5 days to assess the effects on follicle development. Downregulation of E-cad expression led to primordial follicles loss, whereas upregulation of E-cad accelerated follicle growth. Arrowheads indicate primordial follicles, and arrows indicate growing follicles. **e**, **f** Ovarian growing follicle and oocyte counting results showed that knockdown of *E-cad* suppressed follicle activation and decreased the number of oocytes compared with that in the control, whereas overexpression of *E-cad* resulted in a comparable number of total oocytes and growing follicles as in the respective control (refer to Table S4). The experiments were repeated at least three times, and representative images are shown. The data are presented as the means ± S.D. and considered statistically significant at *P* < 0.05. Scale bars: 50 μm

To study the function of E-cad in sustaining the PF pool and activating PFs in the neonatal ovary, an in vitro ovarian culture system was established (Fig. S1a). The counting results showed a comparable number of both total oocytes and growing follicles in 2 dpp ovaries cultured for 5 days in vitro (equal to 7 dpp), and the in vivo developed ovaries at 2 dpp and 7 dpp, respectively (Fig. S1b, S1c), indicating that the in vitro system successfully mimicked the development of follicles in vivo during the early stage. Next, we designed lentivirus constructs expressing *E-cad* shRNA (*E-cad-*KD) and *E-cad* mRNA (*E-cad*-OE) with a GFP reporter to either knockdown or overexpress *E-cad* in neonatal ovaries. The successful transfection efficiency was confirmed by the strong green fluorescent signal observed under fluorescence microscopy after 2 days of culture (Fig. 2a). Accordingly, compared with those in the control ovaries, the mRNA and protein levels of endogenous *E-cad* were efficiently downregulated in *E-cad*-KD injected ovaries and upregulated in *E-cad*-OE injected ovaries (Fig. 2b, c). The treated ovaries were cultured for an additional 3 days and the histological analysis showed that knockdown of *E-cad* significantly reduced the number of primordial and growing follicles in vitro, whereas overexpression of *E-cad* accelerated follicle growth (Fig. 2d, arrows). The quantitative results showed that the number of total oocytes and growing follicles significantly decreased in *E-cad*-KD ovaries, but no remarkable changes were observed in *E-cad*-OE ovaries (Fig. 2e, f). These results indicate that E-cad is involved in the maintenance of PFs and supports the early growth of oocytes in mice.

### Knockdown of *E-cad* triggers oocyte apoptosis

**Fig. 3:**
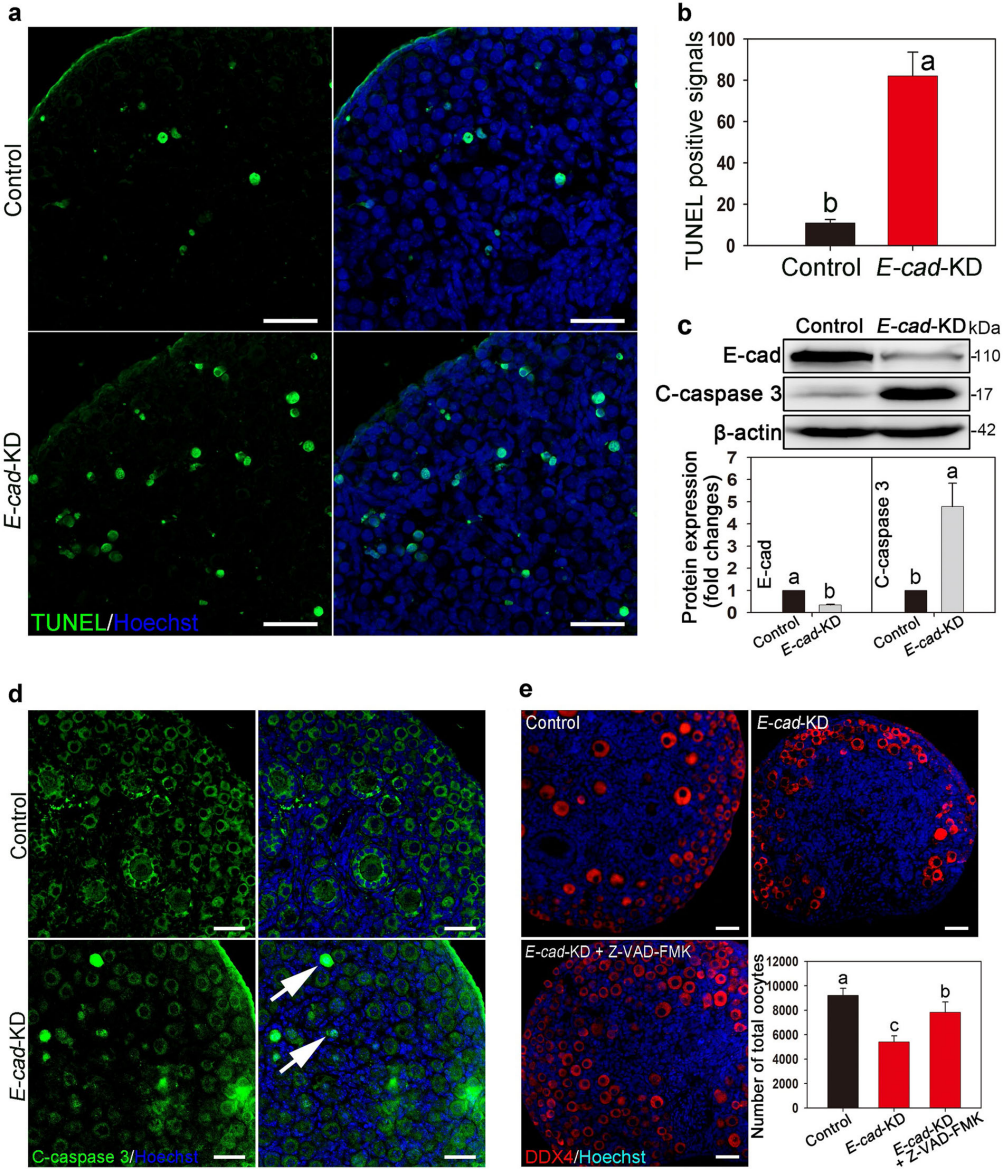
Knockdown of *E-cad* leads to oocyte apoptosis in neonatal mouse ovaries. **a** The ovaries at 2 dpp were transfected with *E-cad*-KD or empty lentivirus and cultured for 3 days. *E-cad*-KD ovaries exhibited more TUNEL-positive signals (green) than the control. **b** Based on the analysis of the five largest cross-sections of cultured ovaries, the average number of apoptotic signals was greater in *E-cad*-KD ovaries (82.1 ± 11.5) than that in the control ovaries (11.0 ± 1.6). **c** Western blot and the relevant intensity analyses revealed that the expression of cleaved caspase 3 (C-caspase 3) was significantly elevated in *E-cad*-KD treated ovaries compared with that in the control group. **d** Immunofluorescence assay showed that knockdown of *E-cad* induced cleaved caspase 3 expression (arrows) in the oocyte nuclei of primordial follicles. **e** Histological and statistics analyses showed that inhibition of caspase activity by Z-VAD-FMK partially rescued the oocyte loss in *E-cad*-KD treated ovaries. Oocytes were labeled with DDX4 (red). The experiments were repeated at least three times, and representative images are shown. The data are presented as the means ± S.D. and considered statistically significant at *P* < 0.05. Scale bars: 50 μm

To investigate whether *E-cad*-KD resulted in significant follicle loss, terminal deoxynucleotidyl transferase-mediated deoxyuridine triphosphate (TUNEL) assay were performed in 2 dpp ovaries cultured for 3 days after lentiviral transfection. The results showed that more TUNEL-positive signals (green) existed in the ovaries treated with *E-cad*-KD than in the control ovaries (Fig. 3a, b). The western blot results showed that the expression of cleaved caspase 3 (the ultimate apoptosis effector) was significantly elevated after knockdown of *E-cad* (Fig. 3c). According to the immunofluorescence staining results, cleaved caspase 3 (green) was localized to the oocyte nuclei in the PFs (Fig. 3d, arrows). Inhibiting caspase activity with Z-VAD-FMK, which is an irreversible general caspase inhibitor, partially rescued the oocyte loss in *E-cad*-KD ovaries (Fig. 3e). These data demonstrate that *E-cad* knockdown-induced follicle loss is caused by oocyte apoptosis.

### E-cad might regulate the maintenance of PFs through NOBOX

**Fig. 4:**
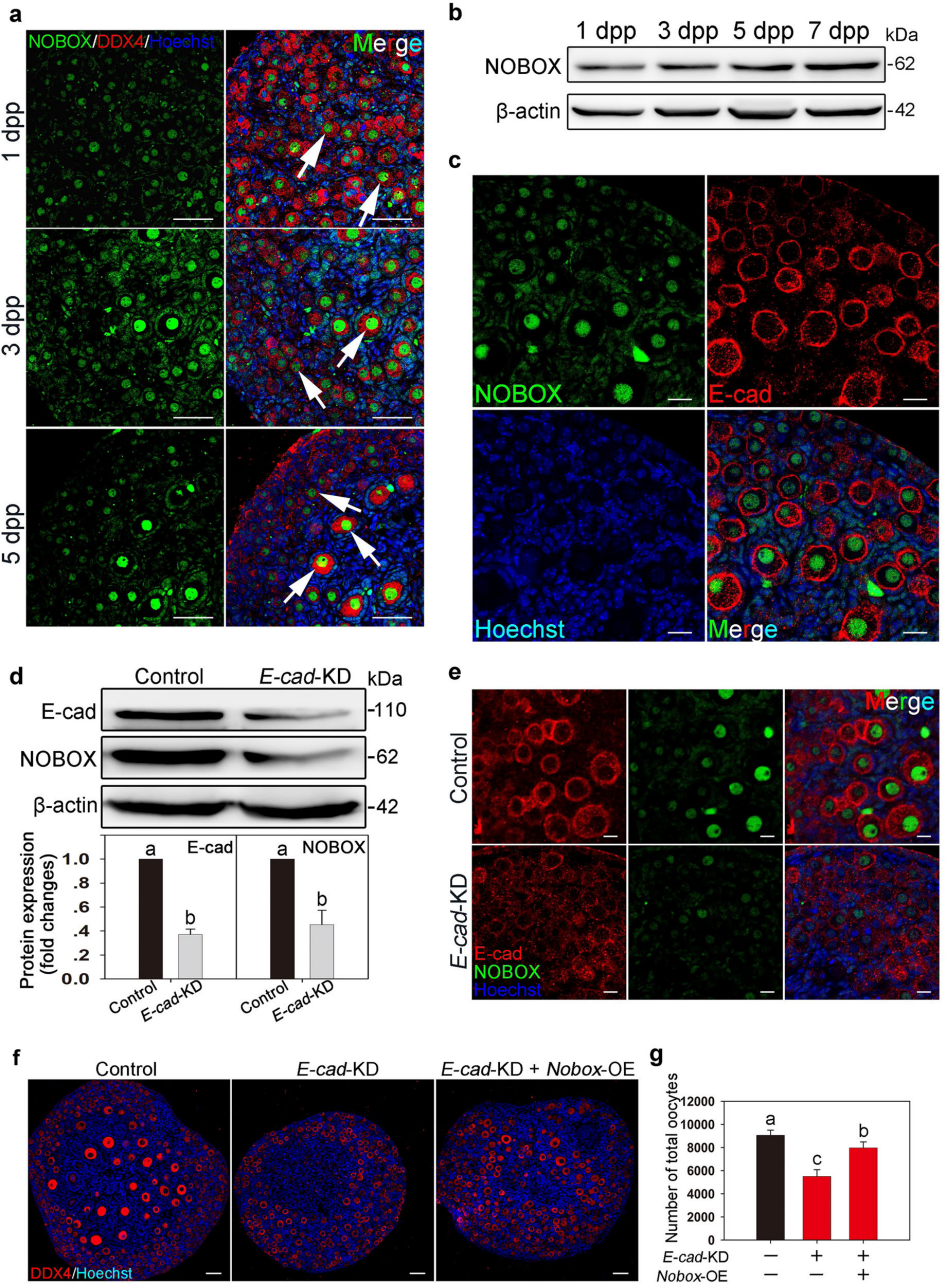
E-cad is responsible for maintaining the primordial follicle pool by regulating NOBOX expression in oocytes. **a** Immunofluorescence staining showed that the expression of NOBOX (green) was specifically localized to the nuclei of all germ cells and that NOBOX expression is increasing with follicle formation and activation (arrows). Oocytes were stained with DDX4 (red). The nuclei were dyed with Hoechst counter-stain (blue). **b** NOBOX expression profile in neonatal ovaries. The expression of NOBOX in the ovary increased over time from 1 dpp to 7 dpp. **c** NOBOX and E-cad were colocalized to the oocytes of ovaries at 3 dpp. NOBOX (green) and E-cad (red) expression levels were elevated in the oocytes of primordial and activated follicles. **d**, **e** Knockdown of *E-cad* suppressed NOBOX expression as shown by western blot assay **d** and immunofluorescence staining **e**. The nuclei were dyed with a Hoechst counter-stain (blue). **f**, **g** Histological and oocyte counting analyses showed that overexpression of *Nobox* in *E-cad*-KD transfected ovaries partially rescued the loss of oocytes compared with the ovaries transfected with *E-cad*-KD alone (refer to Table S5). The experiments were repeated at least three times, and representative images are shown. The data are presented as the means ± S.D. and considered statistically significant at *P* < 0.05. Scale bars: **a**, **f** 50 μm; **c**, **e** 20 μm

Previous and current studies have revealed that E-cad is a transmembrane protein expressed in the oocytes of the rodent ovary, which functions in cyst breakdown and PF formation^24,26,27^. NOBOX is one of the few oocyte-derived transcription factor in follicular growth and oocyte death^13,28,29^, which is consistent with the phenotype observed in the *E-cad*-manipulated ovaries. To further investigate the underlying mechanisms by which E-cad regulates the maintenance of PFs and the early growth of oocytes, the relationship between E-cad and NOBOX in oocytes was studied. The clear nuclei localization of NOBOX was observed in the germ cell cysts, and the expression of NOBOX gradually increased in primordial and growing follicles (Fig. 4a, arrows). The western blot analysis showed that the level of NOBOX in the neonatal mice ovary was increasing from 1 dpp to 7 dpp (Fig. 4b), and these results were confirmed by immunofluorescence. Importantly, NOBOX and E-cad colocalized in the oocytes of primordial and growing follicles (Fig. 4c).

The relationship between E-cad and NOBOX was also studied by interfering with the expression of proteins within the ovaries after birth. The immunoblotting results revealed that knockdown of *E-cad* remarkably suppressed the level of NOBOX compared with that in the control (Fig. 4d). Moreover, NOBOX expression was decreased in the oocytes of *E-cad*-KD transfected ovaries (Fig. 4e). To further confirm the potential regulatory role of E-cad in NOBOX expression, a lentivirus constructs expressing *Nobox* mRNA (*Nobox*-OE) was designed and co-injected into the *E-cad*-KD transfected ovaries. The co-transfection of *E-cad*-KD and *Nobox*-OE significantly decreased E-cad expression and increased NOBOX expression 2 days after transfection (Fig. S2a). The treated ovaries were cultured for an additional 3 days to assess the effects on follicle development by histological analysis, which revealed that overexpression of *Nobox* partially rescued the follicles loss induced by knockdown of *E-cad* (Fig. 4f). The counting results showed a partial recovery of the number of total oocytes and growing follicles in co-transfected ovaries compared with the number in ovaries transfected with *E-cad*-KD lentivirus alone (Fig. 4g, S2b). These results indicate that the maintenance of PFs and oocyte growth may be related to E-cad-induced NOBOX expression in oocytes.

**Fig. 5:**
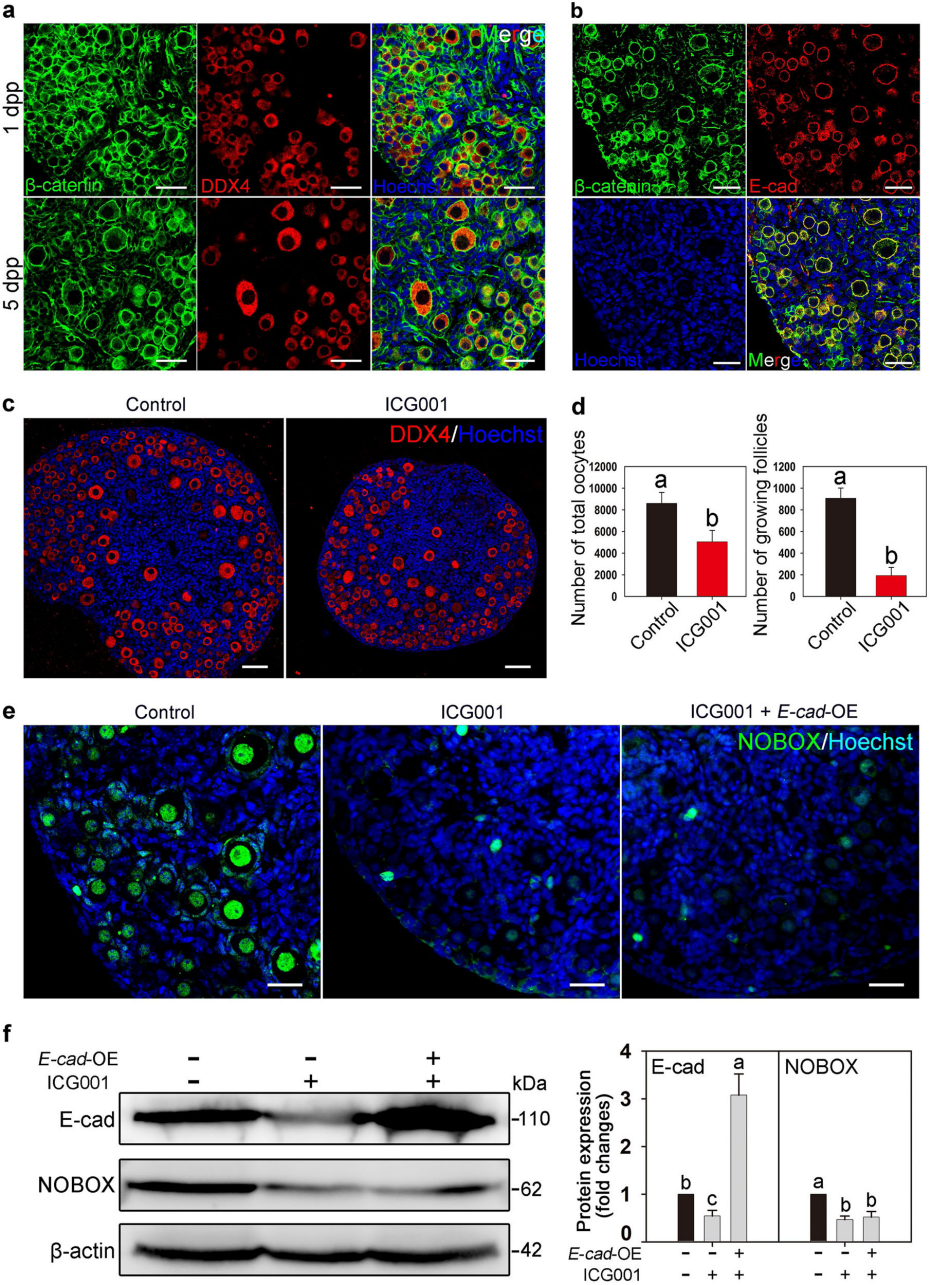
β-catenin mediates E-cad-regulated NOBOX expression. **a** β-catenin expression pattern in neonatal ovaries. β-catenin (green) was ubiquitously expressed in the neonatal ovaries, especially on the cytomembrane of the oocytes in the primordial and growing follicles. **b** β-catenin (green) and E-cad (red) were colocalized in the oocytes of primordial and growing follicles in 3 dpp mouse ovaries. **c**, **d** Inhibiting the transcription activity of β-catenin with ICG001 decreased the number of total oocytes (control, 8459.2 ± 961.0 vs. ICG001, 4855.8 ± 1052.1) and growing follicles (control, 886.7 ± 97.7 vs. ICG001, 182.5 ± 72.0) compared with those in the control. Oocytes were labeled with DDX4 (red). **e**, **f** Expression of NOBOX and E-cad was suppressed in ovaries with the β-catenin inhibitor ICG001 compared with that in the control group, and overexpression of *E-cad* by *E-cad*-OE transfection in ICG001-treated ovaries could not elevate NOBOX expression, as shown by immunofluorescence staining **e** and western blot analysis **f**. NOBOX was stained with green fluorescence and the nuclei were dyed with a Hoechst counter-stain (blue). The experiments were repeated at least three times, and representative images are shown. The data are presented as the means ± S.D. and considered statistically significant at *P* < 0.05. Scale bars: 50 μm

Next, the potential transcription factor that mediates the action of membrane-expressed E-cad on the expression of nuclear localized NOBOX was investigated. β-catenin is a well-identified cytoplasmic molecule that has been shown to interact with E-cad and act as a transcription factor^30,31^. Both oocytes and preGCs in PFs expressed β-catenin (Fig. 5a) and E-cad and β-catenin were colocalized on the oocyte cytomembrane of the follicles (Fig. 5b). After a 5-day in vitro culture of 2 dpp ovaries with the β-catenin selective transcriptional activity inhibitor ICG001, morphological and counting analyses showed a decreased number of PFs compared with that in the control group (Fig. 5c, d). The immunofluorescence and western blot analyses showed that NOBOX expression was reduced in ICG001-treated ovaries compared with that in the control (Fig. 5e, f). Western blot results also showed that E-cad expression was decreased in ICG001-treated ovaries compared with that in the control group (Fig. 5f). However, overexpression of *E-cad* could not rescue the expression of NOBOX in ICG001-treated ovaries, indicating that E-cad may function as the upstream factor in regulating the activity of β-catenin (Fig. 5e, f). These results indicate that membrane-expressed E-cad might play a role in the regulation of NOBOX expression via the shuttling transcription factor β-catenin in oocytes.

### E-cad is involved in regulating PI3K-AKT pathway activity in oocytes

**Fig. 6:**
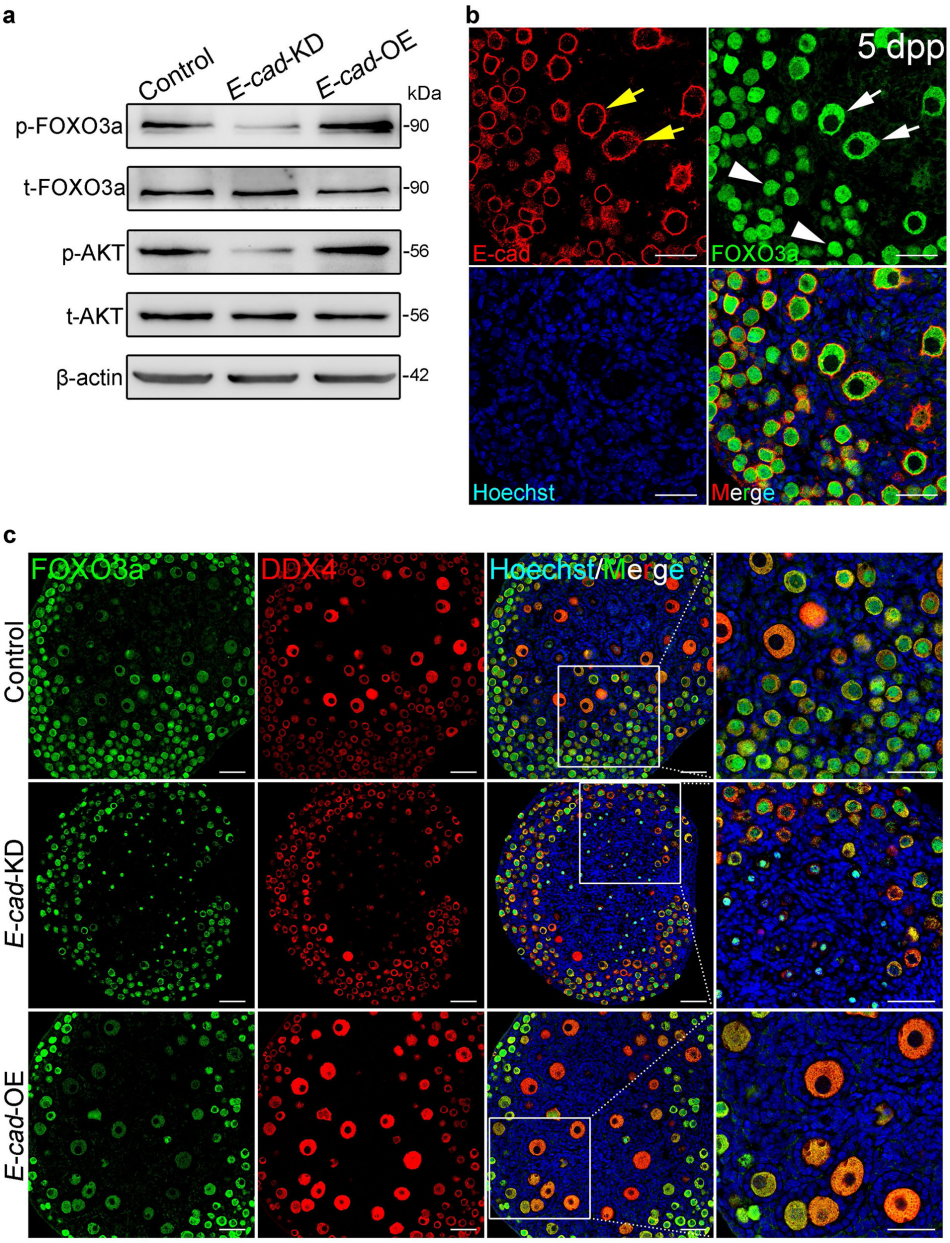
E-cad participates in the maintenance and activation of PI3K signaling. **a** The phosphorylation of both FOXO3a and AKT in the ovaries was affected by E-cad. p-FOXO3a and p-AKT were downregulated in *E-cad*-KD ovaries, but upregulated in *E-cad*-OE ovaries, compared with those in the control. **b** Cellular localization of E-cad and FOXO3a in 5 dpp ovaries. FOXO3a (green) was localized to the nuclei (arrowheads) of the dormant oocytes and translocated to the cytoplasm (arrows) of the activated oocytes in which PI3K signaling was remarkably activated. E-cad (red) expression was increased in the oocytes with cytoplasm-localized FOXO3a. The nuclei were dyed with a Hoechst counter-stain (blue). **c** Immunofluorescence staining showed that the shuttle of FOXO3a from the nuclei to the cytoplasm in the oocytes was either attenuated by *E-cad*-KD transfection or enhanced by *E-cad*-OE transfection compared with the control. The experiments were repeated at least three times, and representative images are shown. Scale bars: 50 μm

To investigate the potential relationship between E-cad and the PI3K pathway, which functions as the dominant signaling pathway in dormant oocyte activation, 2 dpp ovaries were transfected with *E-cad*-KD or *E-cad*-OE lentivirus and cultured for 5 days in vitro. The Western blot analysis showed that the phosphorylation of FOXO3a and AKT was inhibited in *E-cad*-KD transfected ovaries but elevated in *E-cad*-OE transfected ovaries compared with that in the control ovaries (Fig. 6a), indicating that E-cad plays a regulatory role in the activation of the PI3K pathway. The immunofluorescence results showed that FOXO3a shuttled from the nucleus (Fig. 6b, white arrowheads) to the cytoplasm (Fig. 6b, white arrows) in the oocytes with a higher expression of E-cad (Fig. 6b, yellow arrows). In addition, knockdown of *E-cad* suppressed the cytoplasmic expression of FOXO3a, while the FOXO3a sub-cellular localization was intact in *E-cad* overexpression group (Fig. 6c). These results indicate that E-cad is indispensable for activating the PI3K pathway.

## Discussion

Primordial follicles are formed early after labor in mammalian females and may stay dormant for a long time before being recruited into the growing pool^2^. Because PFs are the non-renewable source of fertilizable ova, the survival of PFs is important for the female reproductive lifespan. However, the mechanism by which PFs retain quiescent for decades instead of becoming activated or undergoing progressed apoptosis remains unknown so far. In this study, we demonstrated that E-cad has an important role in sustaining PF survival in neonatal mouse ovaries, which is achieved by regulating NOBOX expression in oocytes and sustaining cell–cell adhesion between germ cells and PF granulosa cells.

NOBOX is among the most important proteins directing oocyte development. However, distinct mechanisms may regulate NOBOX expression in oocytes at different developmental stages. We have recently reported that during PF formation, the expression of NOBOX is regulated by Rac1, which facilitates transcription factor STAT3 importing to the nucleus and binding to the promoter of *Nobox*^32^. In this study, however, E-cad regulated *Nobox* expression by another shuttling protein β-catenin in PFs, which is important for the survival of PFs in cultured ovaries in vitro. In mice, NOBOX is expressed in oocytes as early as 13.5 dpc, and NOBOX expression steadily increases in germ cysts, germ cells in PFs and growing follicles^13^. The deletion of *Nobox* gene generally results in delayed PF formation and oocyte loss after PF formation. NOBOX is an important transcription factor in the early stage of oocyte development as some oocyte-specific genes and follicle development related genes, such as *Mos, Oct4, Pou5fl, Gdf9,* and *Bmp15*, are all directly or indirectly regulated by NOBOX^13,33^.

We found that NOBOX is regulated by E-cad, which interacts with β-catenin after PF pool establishment, implying that β-catenin performs versatile functions in ovaries. As the indispensable member of the canonical WNT/β-catenin pathway, the cell-type-dependent and developmental stage-specific function of β-catenin is determinative for female fertility^34,35^. In somatic cells, active β-catenin signaling is necessary for maintaining female gonad development, but overactivation of β-catenin signaling may be destructive for gametogenesis in germline cells^36,37^. In addition, the stabilization and nuclear localization of β-catenin in primitive germ cells causes germ cell deficiency with delayed cell cycle progression^38^. Our unpublished data also indicate that a delicately balanced β-catenin signaling in oocytes, which is negatively controlled by glycogen synthase kinase 3 beta activity in the fetal ovary, is pivotal for early oogenesis in mice. However, the relationship between E-cad/β-catenin and Rac1/STAT3 induced *Nobox* expression at different follicle developmental stages needs further study. Meanwhile, we found that E-cad expression was partially recovered in the ovaries co-transfected with both *E-cad*-KD and *Nobox*-OE lentiviruses compared with the ovaries transfected with *E-cad*-KD alone, implying that nuclear localized NOBOX might regulate E-cad expression and cell–cell contact in the early stage of folliculogenesis as well.

The cell–cell contacts before and after PF formation could be complex and indispensable. We have previously demonstrated that E-cad between oocytes in the cyst is important for maintaining the structure of cyst and that gap junction contacts established between pregranulosa cells facilitate PF formation^24,39^. As PF is an independent and major unit in the postnatal ovary containing an oocyte and the surrounding pregranulosa cells, it is necessary to clarify the potential role of cadherins in the establishment of cell–cell contacts in PFs. According to our results, cadherins may facilitate oocyte-pregranulosa cell adhesion to maintain the structure of PFs.

Cadherins may function cooperatively to regulate oocyte-pregranulosa cell contacts through heterophilic interactions and serve as anchor proteins to maintain the adherence of pregranulosa cell to oocyte membranes. It has been reported that E-cad regulates cell–cell adhesion through homophilic or heterophilic interactions with neighboring cells^19^. In the current study, the expression of E-cad was elevated on the membrane of oocyte once the PF was formed, suggesting that the cell–cell adhesive contacts was established within the PF. Simultaneously, β-catenin, which is a well-accepted binding protein of E-cad in cell adhesion, colocalized with E-cad on the membrane of oocytes, indicating that this complex plays an important role in maintaining the structure of PFs. However, no comparable expression of E-cad was observed in the surrounding pregranulosa cells in the PFs, whereas β-catenin was steadily expressed in the pregranulosa cells. The immunofluorescence staining showed the clear localization of N-cad, which is another cadherins, in oocytes and surrounding pregranulosa cells in the PFs (Fig. S3a), confirming the previous results observed in rat ovaries^26^. The western blol results also showed an increasing expression of N-cad with follicle development in neonatal ovaries (Fig. S3b). The N-cad expression pattern highlights its potential role in the regulation of β-catenin in somatic cells. In addition, in 8-week-old mice, both E-cad and N-cad were stably expressed in PFs from adult mouse ovaries, suggesting they function persistently after puberty (Fig. S3c). Since E-cad is also expressed in human fetal ovaries^40^, further work is needed to clarify whether E-cad controls the maintaining of PFs in human females. Moreover, a recent study has successfully reconstituted PFs in vitro from mouse pluripotent stem cells and generated fully potent mature oocytes. However, the efficiency of this process is low and the underlying mechanism of directed differentiation and recognition between germ cells and granulosa cells is poorly understood^41^. Our results may provide clue for elucidating this mechanism and improving the efficiency of the in vitro reconstitution of PFs from stem cells.

According to the current study, E-cad may be involved in PF activation. E-cad may facilitate oocyte growth through PI3K signaling after the activation of follicles. PI3K signaling is known as the dominant pathway that controls oocyte growth. Deletion of *Pten*, a major negative regulator of PI3K signaling, results in the excessive activation and premature depletion of PFs^42^. In F9 and breast carcinoma cells, E-cad mediates cell–cell contacts and increases PTEN expression and stability^43,44^. In ovarian cancer cells, E-cad upregulates ligand-independent EGFR trans-phosphorylation, leading to AKT activation^45^. In our study, E-cad may be indispensable for PI3K signaling activation and oocyte growth. The detailed relationship between E-cad and PI3K signaling in PF activation needs further investigation.

In conclusion, our results suggest that oocyte-derived E-cad plays a key role in sustaining PFs by regulating NOBOX expression in mice and cell–cell contacts. These findings provide new insight into the mechanisms of PF pool maintenance and diseases such as POI.

## Materials and methods

### Animals

All CD1 mice were purchased from Beijing Vital River Laboratory Animal Technology Co., Ltd. (Beijing, China) and maintained in mouse facilities that met the requirements of the China Agricultural University Institutional Animal Care and Use Committee. The mice were housed in China Agricultural University under a 16/8 h light/dark cycle at 26 °C and had free access to food and water (Rat & Mouse Maintenance Diet 1022, HFK Bio-tech, China). Female mice (6–8 weeks old) were mated with males overnight to induce pregnancy. Mice with vaginal plugs the following morning were considered 0.5 days post coitus (dpc). The day after partum was considered 1 dpp. The animal experiments were approved by the China Agricultural University Institutional Animal Care and Use Committee.

### Ovary culture

Neonatal pups were killed by cervical dislocation at the designated time. The pup ovaries were micro-dissected in cold phosphate-buffered saline (PBS) using a stereomicroscope (ZSA302, Coic, Chongqing, China) under sterile conditions. The isolated ovaries were cultured on an insert (PICM0RG50, Millipore, USA) in six-well culture plates (Nest, Jiangsu, China) in 1200 μL of Dulbecco’s Modified Eagle’s Medium/Ham’s F12 nutrient mixture (Gibco, Life Technologies, CA) plus ITS (Sigma,1:100, USA) and penicillin-streptomycin solution at 37 °C, 5% CO_2_ and saturated humidity. The culture medium was exchanged every other day.

### Lentiviral production and ovary infection

The lentiviruses were produced in 293 T cells by co-transfecting 5 μg of pMD2.G, 15 μg of psPAX2, and 20 μg of the transfer vector (pSicoR or pLVX-IRES-ZsGreen1). The *E-cad* knockdown lentivirus was constructed by cloning *E-cad* shRNA (Table S1) into the pSicoR vector. The *E-cad* and *Nobox* overexpression lentiviruses were constructed by cloning the open reading frame of *E-cad* or *Nobox* into the pLVX-IRES-ZsGreen1 vector, respectively. The lentiviruses expressing the indicated shRNA and protein contained a GFP tag. The transfections were performed by Lipofectamine 3000 (Invitrogen, USA) and the transfection medium was replaced 6 h after transfection. The viral supernatant was harvested 24 and 48 h after transfection, purified by 0.45-μm membrane filtration and then centrifuged at 40,000 rpm at 4 °C for 2 hours^46^. The lentiviruses were injected into the ovary by a thin glass needle with a mouthpiece. For each injection, the optimal volume was 0.3 μL per ovary. The lentiviral construct pMD2.G and psPAX2 were obtained from Professor Sheng Cui (China Agricultural University). The lentiviral construct pSicoR and pLVX-IREX-ZsGreen1 were gifts from Professor Haibin Wang (Xiamen University).

### Oocytes and follicles counts

The collected ovaries were fixed in 4% paraformaldehyde (PFA) for at least 6 h, embedded in paraffin, and sectioned serially at 5 μm. After staining with DDX4 and counter-dyed with Hoechst, every fifth section was analyzed for the presence of oocytes. The counting results were summed up and multiplied by five to estimate the total numbers of oocytes and follicles in each ovary. The follicles were distinguished from each other as follows: PF (a single oocyte surrounded by several flattened pregranulosa cells) and growing follicle (an enlarged oocyte surrounded by a mixture of squamous and cuboidal somatic cells or an enlarged oocyte surrounded by cuboidal granulosa cells).

### Western blot assay

The collected ovaries were homogenized in WIP Tissue and Cell lysis solution containing 1 mM phenylmethylsulfonyl fluoride (PMSF, Cell Signaling Technologies, USA). The total lysate was centrifuged at 14,000 rpm at 4 °C for 15 min. The supernatant was collected as the total proteins. The protein concentration was measured by a BCA assay (Beyotime, China). The proteins were prepared according to the manufacturer’s instructions. Samples containing 50 µg of protein were separated by 10% sodium dodecyl sulfate–polyacrylamide gel electrophoresis and transferred to polyvinylidene fluoride membranes (IPVH00010, Millipore, USA). Then, the membranes were incubated overnight at 4 °C with the appropriate primary antibody. The primary antibodies used are listed in Table S2. After rinsing thoroughly with tween-buffered saline with Tween, the membranes were incubated with the secondary antibody (1:5000, ZSGB-BIO, China). The membranes were visualized using a SuperSignal West Pico Chemiluminescent Detection System (Prod 34080, Thermo, USA). β-actin (42 kDa, Sigma, USA) was used as the intrinsic control. Alpha Imager 2200 was used to quantify the relative amount of protein.

### RNA extraction and qRT-PCR

The total mRNA was extracted from eight ovaries in each group by TRIZOL Reagent (Invitrogen, Life Technologies, USA) according to the manufacturer’s protocol. First-strand complementary DNA was generated using 1 µg of total RNA (Promega Reverse Transcription System, Promega, USA). Quantitative-RT-PCR was performed using SYBR Select Master Mix (Applied Biosystems, Life Technologies, USA) and operated by Applied Biosystems 7300 Real Time PCR System (Applied Biosystems, Life Technologies, USA). The data were normalized by *β-actin*. Primers are listed in Table S3.

### Immunofluorescence staining

Fresh separated ovaries were fixed in cold 4% PFA overnight, embedded in paraffin, and sectioned at a thickness of 5 μm. After deparaffinizing, rehydration, and high temperature (95–98 °C) antigen retrieval with 0.01% sodium citrate buffer (pH 6.0), the sections were blocked with agarose dissolving buffer (ADB; 3% bovine serum albumin and 1% normal donkey serum in TBS (0.05 M Tris-HCl pH 7.6 and 0.15 M NaCl)) for 60 min at room temperature and incubated with the primary antibodies for 12–16 h at 4 °C. The primary antibodies used are listed in Table S2. Subsequently, after rinsing thoroughly with PBS, the sections were incubated with Alexa Fluor 488- or 555-conjugated secondary antibody (diluted 1:100, Invitrogen) dissolved in ADB for 2 h at 37 °C. Then, the slides were rinsed with PBS, stained with Hoechst 33342 (B2261, Sigma, USA) for 5 min and sealed in anti-fade fluorescence mounting medium (Applygen, China) with coverslips. The sections were examined and photographed under a Nikon Eclipse 80i digital fluorescence microscope or Nikon A1 laser scanning confocal microscope.

### TUNEL staining

The degree of oocyte apoptosis was measured by TUNEL staining using a Click-iT Plus TUNEL Assay (C10617, Life Technologies, USA). The collected ovaries were fixed in 4% PFA, embedded in paraffin, and sectioned to a thickness of 5 μm. Then, the sections were treated according to the manufacturer’s instructions in the in situ apoptosis detection kit. Cells that had multiple DNA breaks were labeled in situ with Alexa Fluor 488 dyes, indicating that they were undergoing apoptosis at the time of fixation. The nuclei were counter-stained by Hoechst 33342 (B2261, Sigma, USA). The sections were examined and photographed under a Nikon Eclipse 80i digital fluorescence microscope.

### Statistical analysis

All experiments were repeated at least three times, and the values are presented as the means ± SEM. The data were analyzed by *t* test or ANOVA and considered statistically significant at *P* < 0.05.

## Electronic supplementary material

Figure S1

Figure S2

Figure S3

Supplementary figure legends
