# Supplementary figures and images for: Oocyte-derived E-cadherin acts as a multiple functional factor maintaining the primordial follicle pool in mice
Source: Cell Death Dis. 2019 Feb 15;10(3):160. doi: 10.1038/s41419-018-1208-3 (PMC6377673; doi:10.1038/s41419-018-1208-3)

Figure S1

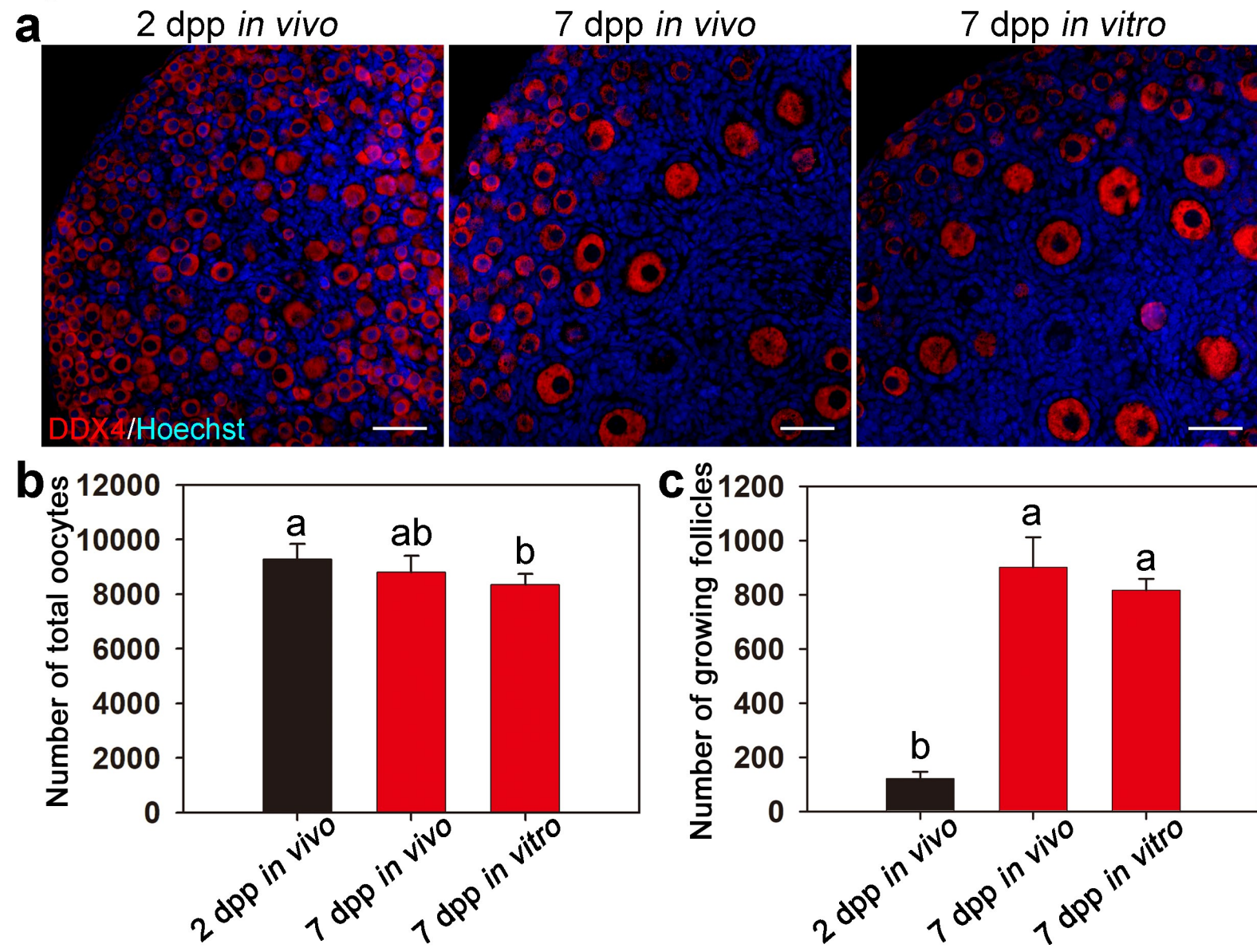

Supplement: Supplementary file 1 — Figure S1 [file 41419_2018_1208_MOESM1_ESM.pdf]

Figure S2

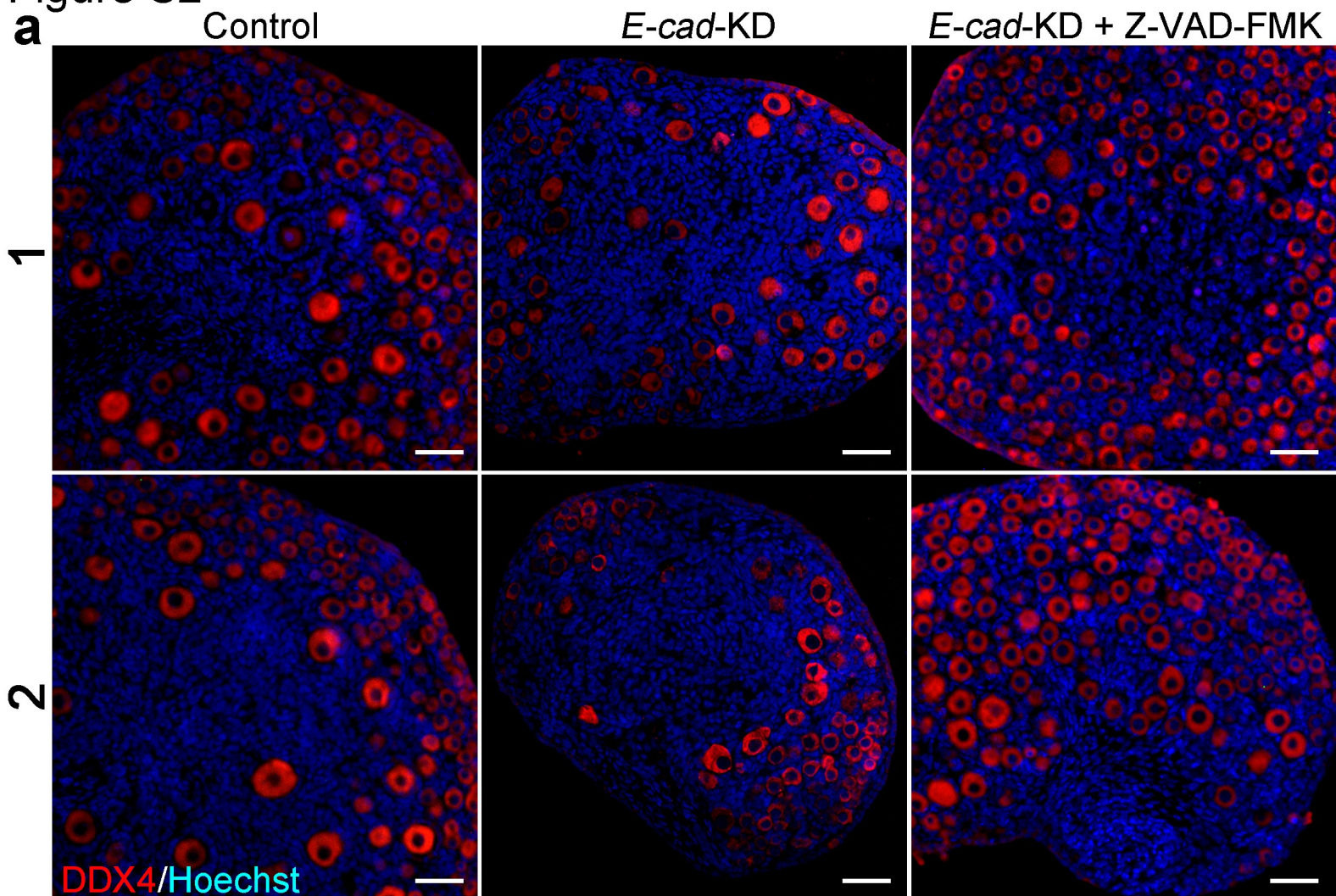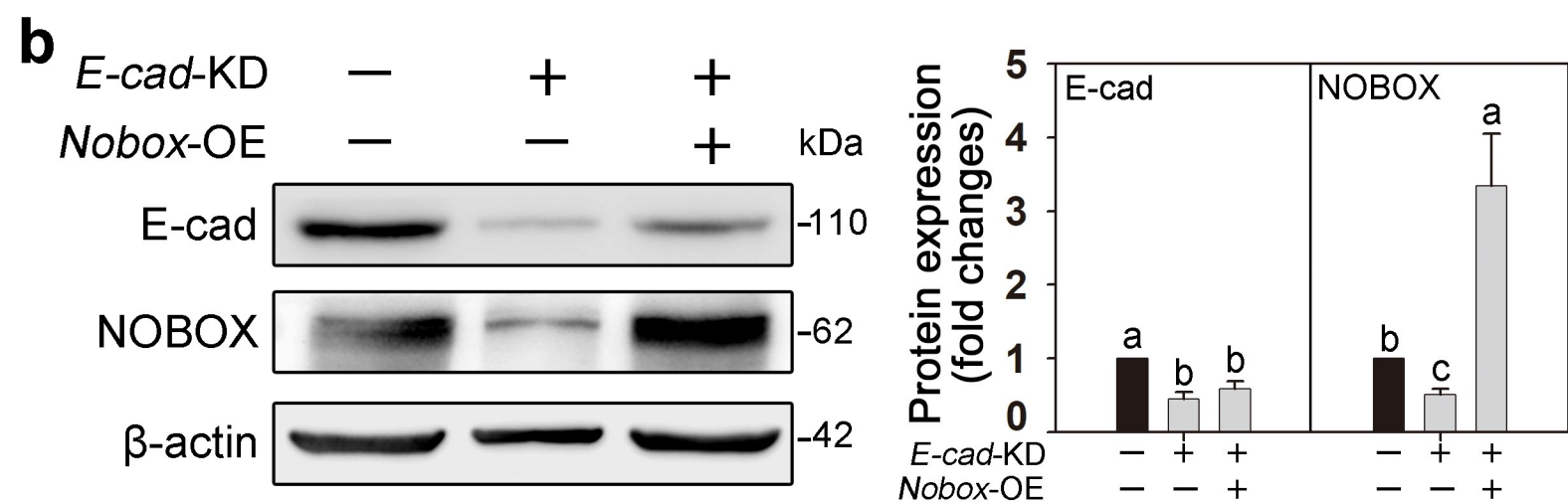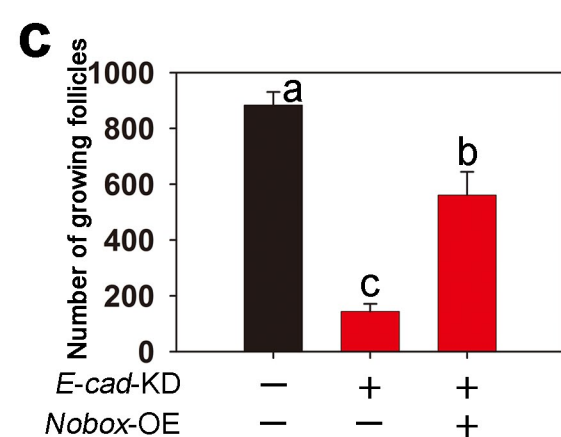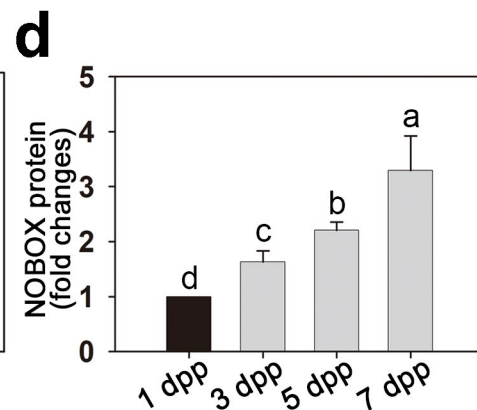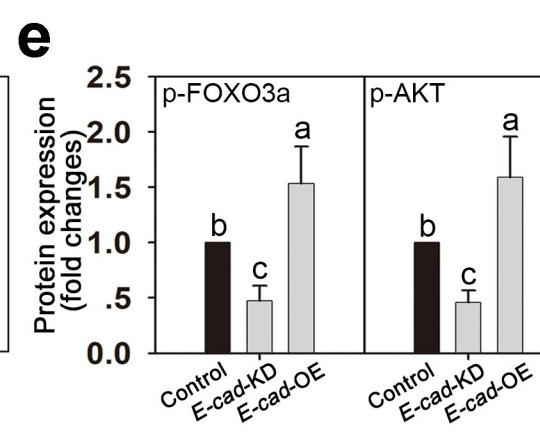

Supplement: Supplementary file 2 — Figure S2 [file 41419_2018_1208_MOESM2_ESM.pdf]

Figure S3

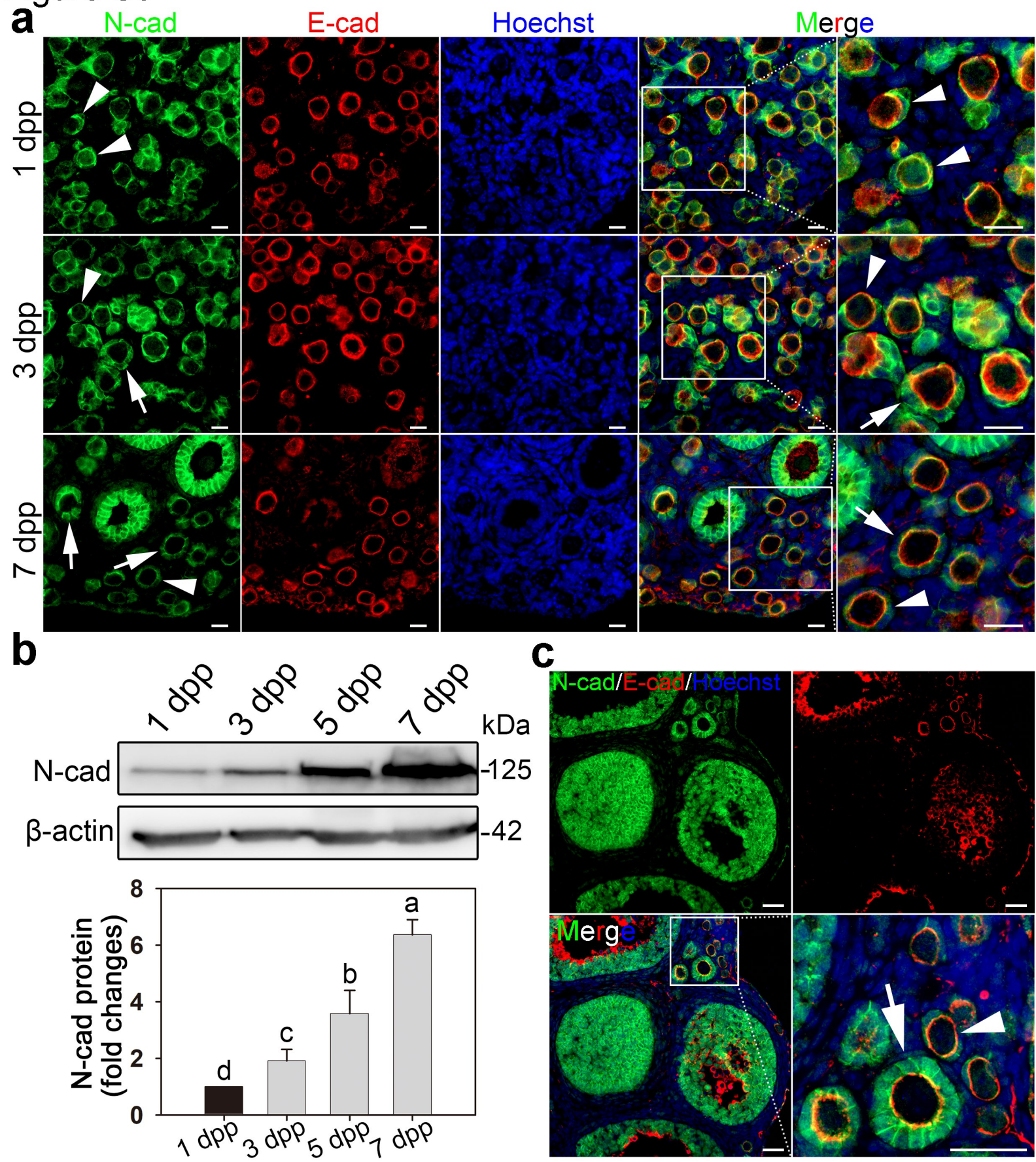

Supplement: Supplementary file 3 — Figure S3 [file 41419_2018_1208_MOESM3_ESM.pdf]
