# Supplementary material for: Oocyte-derived E-cadherin acts as a multiple functional factor maintaining the primordial follicle pool in mice
Source: Cell Death Dis. 2019 Feb 15;10(3):160. doi: 10.1038/s41419-018-1208-3 (PMC6377673; doi:10.1038/s41419-018-1208-3)
Supplement: Supplementary file 4 — Supplementary figure legends [file 41419_2018_1208_MOESM4_ESM.docx]

**Fig. S1** Histological and statistical analyses showed comparable dynamics of follicle maintenance and developmental competency in the ovary in the *in vitro* culture system and the ovary developed *in vivo*.

**a** Immunofluorescence staining of ovaries at 2 dpp and 7 dpp *in vivo* and 2 dpp followed by 5 days of *in vitro* culture (equal to 7 dpp). Oocytes were stained with DDX4 (red), and the nuclei were dyed with a Hoechst counter-stain (blue). **b, c** The total oocytes and growing follicles were quantified in ovaries at 2 dpp, 7 dpp and 2 dpp plus 5 days of *in vitro* culture. A comparable number of both total oocytes (2 dpp *in vivo*, 9292.5 ± 541.6; 7 dpp *in vivo*, 9067.5 ± 484.8; 7 dpp *in vitro*, 8432.5 ± 321.1) and growing follicles (2 dpp *in vivo*, 131.7 ± 24.0; 7 dpp *in vivo*, 849.2 ± 92.4; 7 dpp *in vitro*, 810.8 ± 56.6) were quantified. The experiments were repeated at least three times, and representative images are shown. The data are presented as the means ± S.D. Scale bars: 50 μm.

**Fig. S2**

**a** The ovaries at 2 dpp were transfected with *E-cad*-KD or empty lentivirus and cultured for 5 days. Histological analysis showed that inhibition of caspase activity by Z-VAD-FMK partially rescued the oocyte loss in *E-cad*-KD treated ovaries. Oocytes were labeled with DDX4 (red). Parallel experimental results are shown. **b** Validation of the efficiency of *E-cad* knock-down and *Nobox* overexpression in cultured ovaries. Ovaries at 2 dpp were transfected with the indicated lentivirus or vehicle and cultured *in vitro* for 48 hours. Western blot and the relevant intensity analyses showed that E-cad expression was successfully decreased and NOBOX expression was increased in the relevant lentiviral transfected ovaries compared to the control group. **c** Counting results showed that the number of growing follicles was prominently decreased in *E-cad*-KD treated ovaries but partially recovered in the ovaries treated with both *E-cad*-KD and *Nobox*-OE lentiviruses (refer to Table S5). **d** The intensity analysis of the Western blot results showed that NOBOX expression was significantly increasing with time from 1 dpp to 7 dpp. **e** The intensity analysis of the Western blot results showed that both the expression levels of p-FOXO3a and p-AKT were decreased in *E-cad*-KD ovaries, but increased in *E-cad*-OE ovaries, compared with those in the control. The experiments were repeated at least three times, and representative images are shown. The data are presented as the means ± S.D. and considered statistically significant at P < 0.05. Scale bars: 50 μm.

**Fig. S3**

**a** N-cad expression pattern in neonatal ovaries. N-cad (green) was expressed on the cytomembrane of the granulosa cells in primordial and growing follicles and the cytomembrane of the oocytes in cyst, PFs and growing follicles. Arrowheads indicate primordial follicles, and arrows indicate growing follicles. E-cad was stained with red fluorescence, the nuclei were counter-stained by Hoechst (blue). **b** The expression of N-cad in the ovary increased with time from 1 dpp to 7 dpp. **c** E-cad and N-cad expression pattern in 8-week-old mouse ovaries. Both E-cad (red) and N-cad (green) were expressed in the primordial (arrowhead) and growing follicles (arrow). The nuclei were counter-stained with Hoechst (blue). The experiments were repeated at least three times, and representative images are shown. Scale bars: **a** 20 μm; **c** 50 μm.
